# Quality of recovery and pre-existing impaired cognition in patients undergoing advanced GI endoscopic procedures with patient-controlled sedation: a prospective observational cohort study

**DOI:** 10.1016/j.igie.2023.07.002

**Published:** 2023-07-13

**Authors:** Sara Lyckner, Michelle S. Chew, Andreas Nilsson

**Affiliations:** 1 Department of Biomedical and Clinical Sciences; 3 Department of Anesthesia and Intensive Care, Linköping University, Linköping, Sweden; 2 Department of Anesthesia and Intensive Care, Mälarsjukhuset, Eskilstuna, Sweden

## Abstract

**Background and aims:**

Advanced GI endoscopy (GIE) procedures are common, and many patients undergoing these procedures are elderly with comorbidities. The procedures are commonly conducted in day surgery, and good postprocedural recovery is assumed. How patients recover has been poorly studied, however. The primary aim of this study was to describe the quality of recovery (QoR) in patients undergoing advanced GIE procedures with patient-controlled sedation. A secondary aim was to evaluate if impaired preprocedural cognitive ability was an independent risk for lack of recovery.

**Methods:**

Measurements were conducted by using the 15-item QoR instrument (QoR-15) before the GIE procedure (baseline) and on days 1 and 5 postprocedure. Postprocedural recovery scores were compared versus baseline scores. Lack of recovery was defined as any decrease in the QoR-15 score compared with baseline. Cognitive ability was assessed by using the clock-in-the-box test. A multivariable logistic regression analysis was used to test the independent association between preprocedural cognitive impairment and lack of recovery.

**Results:**

A total of 93 patients were included in the study. The QoR-15 score significantly improved at postprocedure day 1 (*P* = .001) with no further improvement at day 5. One-third of the patients did not recover and even had negative trajectories. Preprocedural impaired cognitive ability was present in 41% of patients but could not independently explain the negative changes in the QoR-15 score.

**Conclusions:**

QoR significantly improved for a majority of patients by day 1 after advanced GIE procedures. However, one-third of patients displayed a lack of recovery. We highlight the need for improved strategies to increase quality of postendoscopic recovery and the recognition of preoperative cognitive impairment.

Advanced GI endoscopy (GIE) procedures, including ERCP and EUS, are often performed as daycare procedures.^1^ Because these procedures cause increased pain and discomfort compared with other GIE procedures,^2^ these advanced GIE procedures are commonly performed with the patient under general anesthesia or heavy sedation. General anesthesia and sedation are considered equally effective and safe in terms of all-cause or anesthesia/sedation-related adverse events.^3^ Also, moderate sedation and endoscopist-directed sedation have been regarded as safe and cost-effective.^4^ Patient-controlled sedation (PCS) for ERCP expedites postanesthesia recovery^5^^,^^6^ and reduces the risk of rescue interventions for sedation-related adverse events.^7^

Because many patients scheduled for advanced GIE procedures are elderly and have multiple comorbidities, peroperative cardiorespiratory homeostasis is crucial for maintaining good postprocedural recovery. Despite a common perception that these patients recover well and are suitable for same-day discharge, the quality of patients’ recoveries is largely unknown. In this regard, PCS may be attractive, as the likelihood of anesthetic over-administration is minimized.^7^ To our knowledge, no study has described quality of recovery (QoR) in patients undergoing advanced GIE procedures with PCS.

Preoperative impaired cognitive function is associated with hospital mortality, adverse events, and length of stay.^8^ Increased age and impairment of activities of daily living are also risk factors for poor postoperative recovery after major surgery.^9^ However, the literature regarding postprocedural recovery after advanced GIE procedures has often focused on the choice of drugs, doses administered, or sedation versus general anesthesia.^10^ At least one-fifth of all elective surgical patients aged ≥65 years exhibit cognitive impairment.^11^ It is therefore conceivable that the advanced GIE population that also consists of older patients with comorbidities may have poor postprocedural recovery, even within the day surgery setting.

The primary aim of the current study was to describe QoR among patients undergoing advanced GIE procedures with PCS up to 5 days postprocedure. A secondary aim was to evaluate if impaired cognitive ability was an independent risk factor for lack of recovery. We hypothesized that QoR was impaired at day 1 with a full recovery to baseline scores at 5 days postprocedure. We also hypothesized that impaired preprocedure cognitive ability was associated with a lack of recovery, defined as any decrease in QoR at day 1 or 5 compared with baseline scores.

## Methods

### Study design and participants

We adhered to the Strengthening the Reporting of Observational Studies in Epidemiology reporting checklist (Supplementary Table 1, available online at www.igiejournal.org).^12^ The study was approved by the Regional Ethical Committee of Linköping (No. 2019–04545) and was registered at ClinicalTrials.gov. Adult patients (aged ≥18 years) undergoing advanced GIE procedures (ERCP or EUS) and receiving PCS at a university hospital in Sweden between November 2019 and July 2020 were eligible for the study. PCS was used in patients undergoing the procedures; a handheld button allowed the patients to titrate their own sedation.

All patients gave their written, informed consent before inclusion and were included consecutively. Exclusion criteria were difficulties in understanding written and spoken language (Swedish) and prior inclusion in the study.

### Data collection

Study nurses at the site interviewed patients at the bedside before the procedure. We used the Swedish version of the 15-item QoR (QoR-15) (Supplementary Table 2, available online at www.igiejournal.org), a validated instrument for the assessment of postoperative quality of recovery,^13^ at 3 time points: preprocedural (baseline) and 1 day and 5 days after the procedures. The instrument measures recovery in 5 domains, and the maximal score is 150 points, with higher scores indicating better recovery. Preprocedural QoR-15 was measured by the study nurse with face-to-face interviews, and follow-ups were made by telephone. The QoR-15 total score and the change in scores between time points were calculated. Lack of postprocedural recovery was defined as any decrease in QoR-15 score compared with baseline.

The clock-in-the-box (CIB) test was used to assess preprocedural cognitive function. CIB is a simple screening test and has been validated for perioperative settings.^14^ CIB testing was performed before the procedures. The patient was allowed some time to read the instructions before being asked to perform the task of drawing the clock. Performance of the drawing was rated on a score from 0 to 8. Impaired cognition was defined as a total score ≤5.

Patient characteristics and perioperative data such as age, sex, American Society of Anesthesiologists physical status classification, cancer, priority of procedure, duration of the procedure (length of surgery), and type of procedure (surgery) were collected from patients’ medical records.

### Statistical analysis

Due to the exploratory nature of this study, no power calculation was performed before initiating the study. The sample size was therefore limited by the inclusion period and was consistent with sample sizes of prior studies in QoR.^15^

Descriptive data are reported as frequency (%) or median (interquartile range). Data distribution was tested by using the Kolmogorov-Smirnov test. For comparison between groups, the Mann-Whitney *U* test was used.

To study the temporal characteristics of QoR-15, data were analyzed by using Friedman’s analysis of variance on ranks with a post hoc Wilcoxon signed rank test. The effect of impaired cognitive ability on lack of recovery was evaluated by using multivariable logistic regression. The covariates were preprocedural impaired cognitive function (CIB score ≤5), duration of surgery, age, and emergency procedure, and the covariates were chosen a priori based on previous literature or clinical plausibility. The outcome was lack of postprocedure recovery, defined as any decrease in QoR-15 score compared with baseline scores. If any individual item was missing from the 15-item questionnaire, these were replaced by the group mean for that item. The degree of missingness for all possible items was <1%.

IBM SPSS software version 25.0 (IBM SPSS Statistics, IBM Corporation, Armonk, NY, USA) was used to perform the statistical analyses. All *P* values <.05 were considered as significant.

## Results

**Figure 1:**
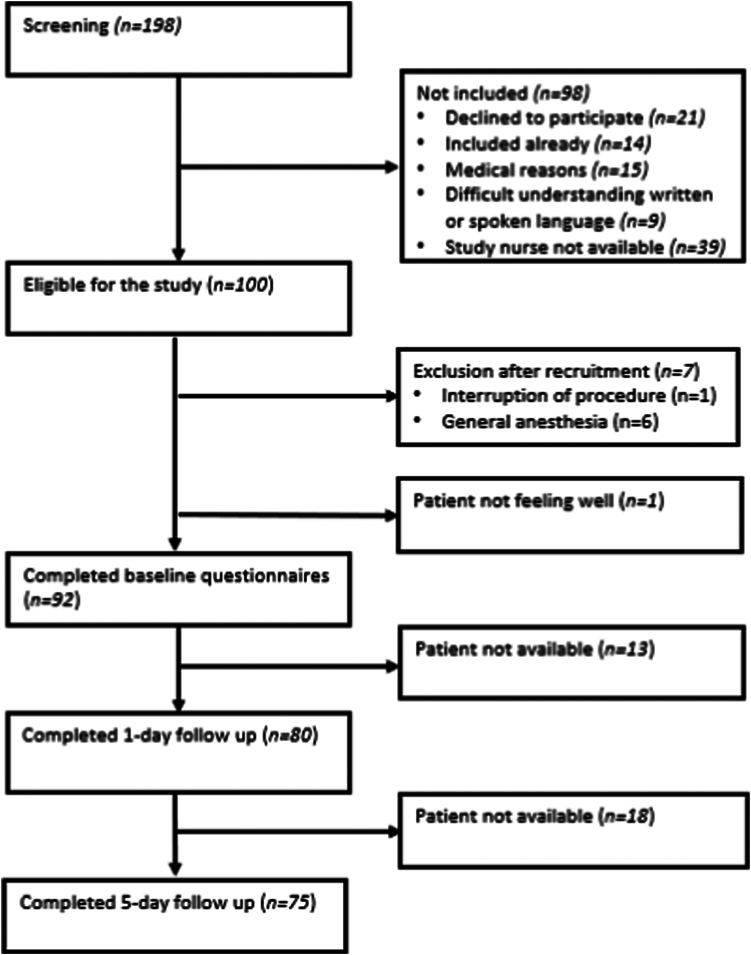
Study flowchart describing screening, exclusion, and inclusion of patients. Total of 93 patients were assigned to the follow-up.

A total of 93 patients were included and assigned to the follow-up group (Fig. 1). Demographic characteristics and clinical data for the study group are presented in Table 1.

**Table 1:** Demographic and clinical data for the study group

Study group (n = 93)	Value
Age, y	69 (58-75)
Female	51%
ASA PS class I/II/III	13%/48%/39%
Type of procedure (ERCP/EUS)	49%/51%
Malignancy	51%
Emergency procedure	35%
Length of surgery, min	35 (22-65)

Values are median (interquartile range) unless otherwise indicated.

*n,* Number of participants; *ASA PS,* American Society of Anaesthesiologists physical status classification.

### Quality of recovery

**Figure 2:**
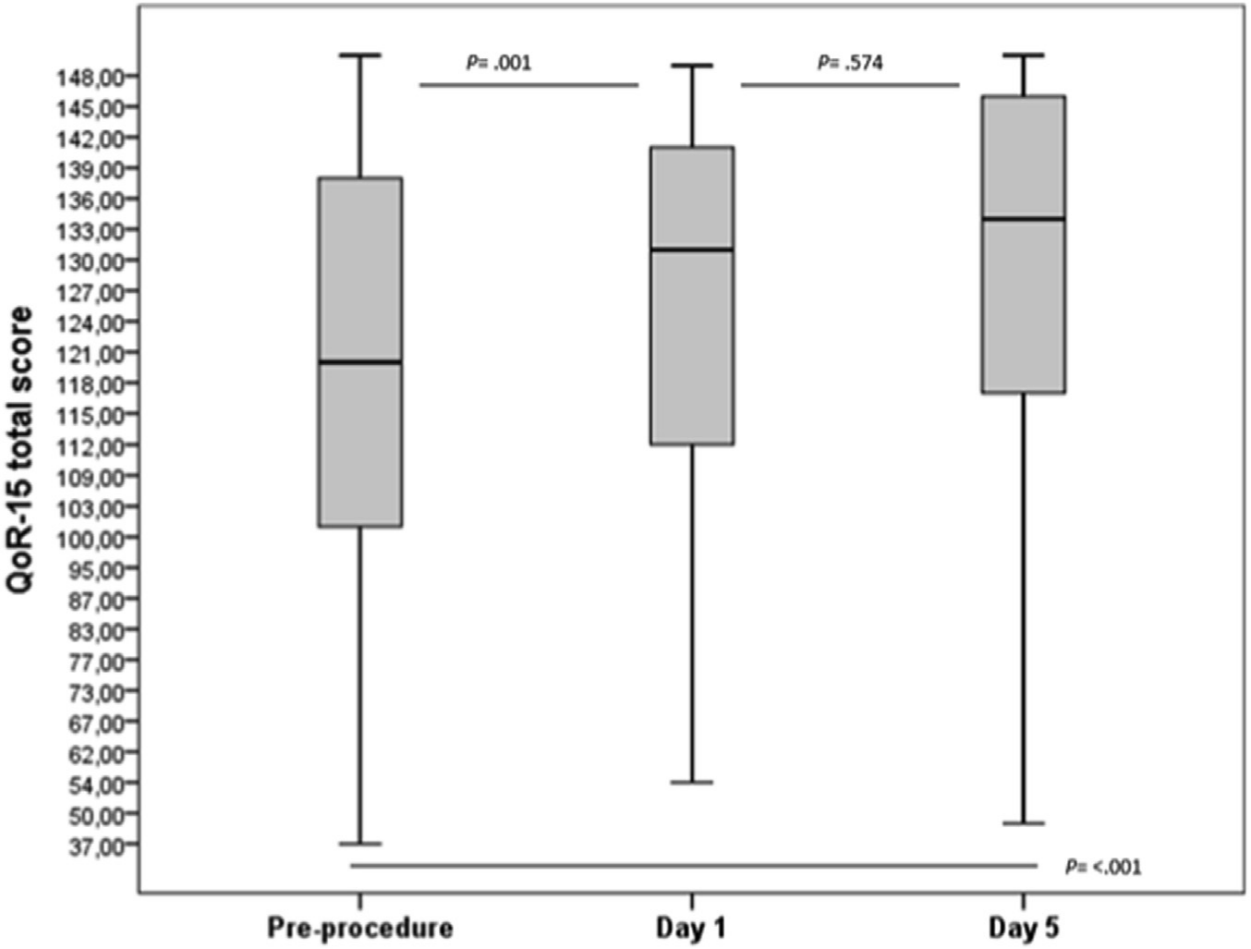
Box plot with median scores for the 15-item quality of recovery tool (QoR-15) for the 3 sampling points.

The QoR-15 score was reduced at the preprocedural assessment and significantly improved postprocedural day 1 (120 [100-138] vs 131 [111-141], *P =* .001). No further improvement was seen at day 5 (131 [111-141] vs 134 [117-146], *P =* .574) (Fig. 2). On the first day of follow-up, 50 patients (63%) had an increase in QoR-15 score and 52 patients (70%) at day 5 compared with baseline. A decrease in QoR-15 total score was seen in 29 patients (37%) at day 1 and 22 patients (30%) at day 5. The median negative change was –9 (–19 to –3) at day 1 and –8 (–22 to –3) at day 5. A subanalysis comparing the 2 main groups, ERCP and EUS, showed no difference in negative change at day 1 or day 5 (Supplementary Table 3, available online at www.igiejournal.org). Data were missing at day 1 follow-up for 13 patients (14%) and at the 5-day follow-up for 18 patients (19%). Patients with a missing follow-up at day 5 had a lower QoR-15 total score at day 1 (*P =* .049) (data not shown).

**Supplementary Figure 1:**
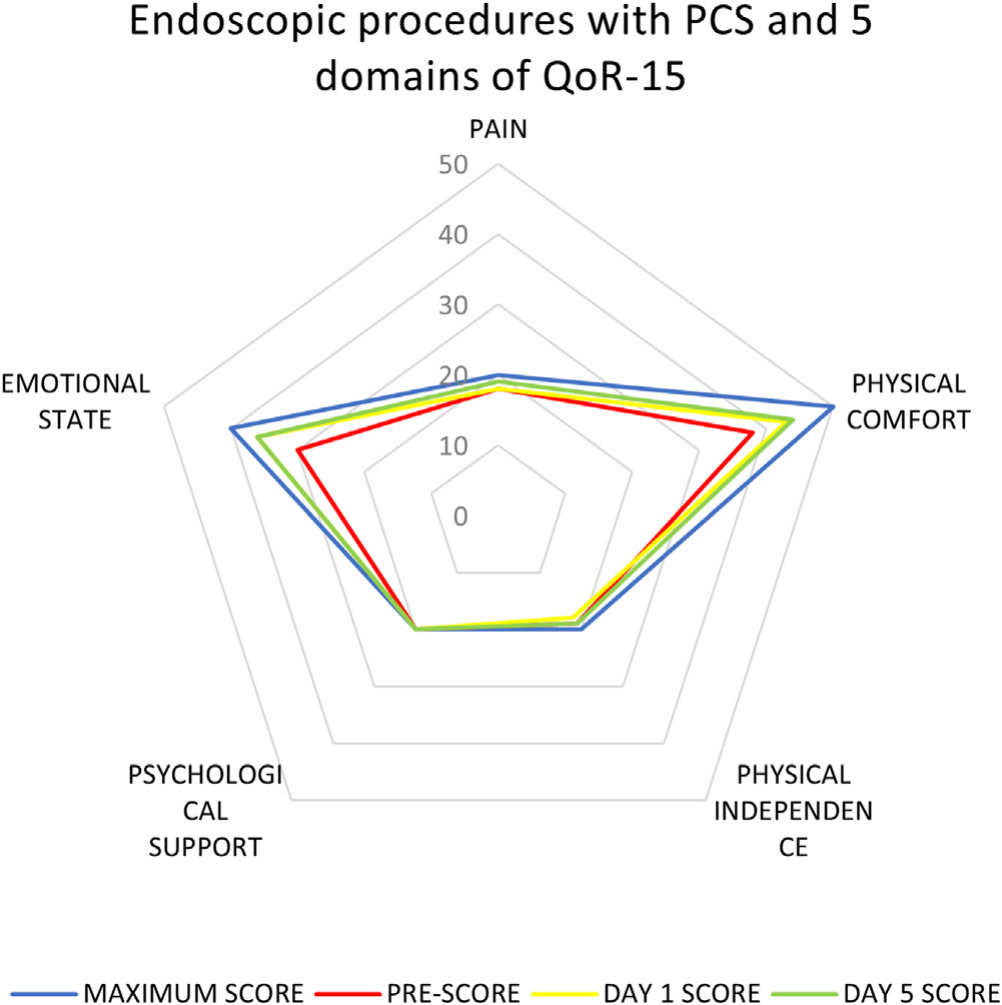
The 15-item quality of recovery tool (QoR-15) and 5 domains at the 3 sampling points. *PCS*, Patient-reported score.

The median QoR-15 scores for each item at the 3 different time points were calculated, and the lowest scores were seen in items “good sleep,” “feeling rested,” and “general well-being” occurring at the preprocedural sampling point baseline and continuing to day 5. Items “worried or anxious,” “sad or depressed,” “moderate pain,” and “severe pain” showed significant improvement at days 1 and 5 compared with preprocedural values (Table 2). Overall, the most affected domains for QoR-15 were pain, physical comfort, and emotional state (Supplementary Fig. 1, available online at www.igiejournal.org).

**Table 2:** Median QoR-15 scores (median [IQR]) per item at 3 sample points: differences measured from baseline to day 1 and day 5

QoR-15 item	Domain	Baseline (n = 92)	Day 1 (n = 80)	Day 5 (n = 75)	*P* value∗
Able to breath easily	Physical comfort	9.5 (8-10)	10 (9-10)^a^	10 (8-10)^b^	^a^ .010 ^b^ .104
Been able to enjoy food	Physical comfort	9.0 (6-10)	9.0 (5-10)^a^	9.0 (6-10)^b^	^a^ .243 ^b^ .124
Feeling rested	Physical comfort	7.5 (4-9)	7.0 (5-8)^a^	8.0 (6-10)^b^	^a^ .260 ^b^ .003
Have had good sleep	Physical comfort	7.5 (4-9)	8.0 (5-10)^a^	8.0 (7-10)^b^	^a^ .011 ^b^ .001
Able to look after personal hygiene unaided	Physical independence	10 (10-10)	10 (10-10)^a^	10 (10-10)^b^	^a^ .055 ^b^ .894
Able to communicate with family and friends	Psychological support	10 (10-10)	10 (10-10)^a^	10 (10-10)^b^	^a^ .472 ^b^ .647
Getting support from hospital, doctors, nurses	Psychological support	10 (10-10)	10 (9-10)^a^	10 (10-10)^b^	^a^ .452 ^b^ .974
Able to return to work or usual activities	Physical independence	10 (6-10)	8.5 (5-10)^a^	10 (5-10)^b^	^a^ .386 ^b^ .538
Feeling comfortable and in control	Emotional state	9.0 (7-10)	10 (8-10)^a^	10 (8-10)^b^	^a^ .015 ^b^ .146
Having a feeling of general well-being	Emotional state	8.0 (4-10)	8.0 (5-9)^a^	8.0 (6-10)^b^	^a^ .135 ^b^ .016
Moderate pain	Pain	8.0 (4-10)	8.0 (5-10)^a^	9.0 (6-10)^b^	^a^ .965 ^b^ .240
Severe pain	Pain	10 (6-10)	10 (10-10)^a^	10 (10-10)^b^	^a^ .041 ^b^ .036
Nausea and vomiting	Physical comfort	10 (6-10)	10 (10-10)^a^	10 (10-10)^b^	^a^ <.001 ^b^ .041
Feeling worried or anxious	Emotional state	8.0 (3-10)	10 (7-10)^a^	10 (7-10)^b^	^a^ .001 ^b^<.001
Feeling sad or depressed	Emotional state	8.5 (5-10)	10 (7-10)^a^	10 (8-10)^b^	^a^ .003 ^b^<.001
Total score		120 (100-138)	131 (111-141)^a^	134 (117-146)^b^	^a^ .001 ^b^<.001

*QoR-15*, 15-Item quality of recovery tool; *IQR*, interquartile range; *n,* number of participants.

∗ *P* = Wilcoxon signed rank test.

### Cognitive screening

Preprocedural impaired cognitive ability was present in 38 patients (41%). When comparing patients with and without impaired cognitive ability, no differences were seen in QoR-15 score at any time point (Table 3).

**Table 3:** Demographic and clinical data for the group with impaired cognition (CIB score ≤5) compared with the group with no impaired cognition (CIB score >5)

Study group parameter	CIB score ≤5 (n = 38)	CIB score >5 (n = 54)	*P* value∗
Age, y	69 (63-78)	67 (52-73)	.054
Female	45%	54%	.400
ASA PS class I/II/III	8/42/50	17/54/29	.044
Type of procedure (ERCP/EUS)	40/60	57/43	.092
Malignancy	57	46	.247
Emergency procedure	31	39	.371
Length of surgery, min	31 (20-59)	40 (29-71)	.07
Preoperative QoR-15 score	122 (103-139)	115 (100-136)	.282
Postoperative QoR-15 day 1 score	131 (113-142)	133 (111-142)	.980
Postoperative QoR-15 day 5 score	138 (122-145)	132 (116-146)	.553
Preoperative CIB score	4 (2-5)	7 (6-8)	<.001

Values are median (interquartile range) unless otherwise indicated.

*CIB*, Clock-in-the-box cognitive screening test; *n*, number of participants; *ASA PS*, American Society of Anesthesiologists physical status classification; *QoR-15*, 15-item quality of recovery tool.

∗ *P* = Mann-Whitney *U* test.

Impaired cognitive ability was not an independent predictor of lack of recovery at day 1 (*P* = .088) or at day 5 (*P* = .973) (Table 4).

**Table 4:** Logistic regression including risk factors and cognitive impairment defined as CIB score ≤5

Risk factor	Lack of postoperative recovery on day 1			Lack of postoperative recovery on day 5		
	aOR	95% CI	*P* value	aOR	95% CI	*P* value
Cognitive impairment	2.481	.873-7.055	.088	1.019	.350-2.962	.973
Emergency procedure	.371	.122-1.134	.082	.694	.229-2.106	.519
Length of surgery	1.020	1.002-1.038	.025	1.006	.990-1.023	.466
Age	.995	.960-1.032	.805	1.031	.990-1.073	.138

Lack of postoperative recovery is defined as any negative change in the 15-item quality of recovery score compared with baseline.

*CIB*, Clock-in-the-box cognitive screening test; *aOR*, adjusted odds ratio; *CI*, confidence interval.

## Discussion

The current study reported 3 major findings: first, QoR significantly improved in most of the patients, with early recovery shown at postprocedural day 1. However, one-third of the patients had not recovered by day 1 and day 5. Instead, their recoveries even had negative trajectories. Second, the most positively affected domains of QoR-15 were pain, physical comfort, and emotional state, whereas psychological support and physical independence remained unchanged throughout the study period. Third, impaired cognitive ability was present in 41% of the patients but did not explain the lack of postprocedural recovery.

Early recovery was generally observed among patients undergoing advanced GIE procedures, consistent with previous studies in day surgical patients undergoing orthopedic (n = 663) or laparoscopic (n = 73) surgery.^16^^,^^17^ The median improvement was 13 points on day 1 postprocedure. This is considerably more than what has previously been defined to be a minimal clinically important difference score (6 or more) that would imply a meaningful change in health status.^18^ For example, patients with pancreatic obstruction rapidly feel better within hours after treatment, and the QoR-15 is suggested to be sensitive enough to measure this recovery. Nevertheless, our data also showed that one-third of patients had not fully recovered at day 1 or day 5, and QoR-15 scores were lower than at baseline. Comparable data are limited, but incomplete recovery for 1 of 3 patients at day 1 after colonoscopy has been reported.^19^ ERCP is a more complex procedure, usually takes a longer time to perform, requires both sedatives and analgesics, and some patients are heavily burdened with comorbidities, compared with other GIE procedures,^20^ and these factors may explain some of the findings in nonrecovered patients.

Although overall QoR was good, several notable findings were observed within individual domains. Emotional state and physical comfort improved postoperatively, which was expected. Psychological support and physical independence did not change through the study period. Low values on psychological support and physical independence may reflect that the GIE procedures represent only one step of many for patients with cancer and critical illness. Additional data from patient interviews may improve understanding of ongoing needs for this group of patients.

The timing in measurement of QoR must be considered to determine the lowest postprocedural dip. Myles et al^21^ suggested that an accurate time for QoR in day care may be just before discharge, but this does not capture the lowest point in postoperative recovery. The physical and psychological imprint from advanced GIE procedures may be at its largest just hours afterward, and this period could be used for the identification and adjustment of the postprocedure care and follow-up, before discharge. Although the majority of patients had improved QoR-15 scores, lack of recovery was still observed at days 1 and 5 postprocedure. There were low scores or small positive changes in the domains of pain, physical comfort, and emotional state, suggesting that future efforts to improve quality of recovery after advanced GIE procedures should specifically target these areas. Another notable finding was the low preprocedural score, reflecting perhaps the chronic nature of the underlying disease in this population. Low preoperative QoR-15 scores may themselves predict a poor recovery and longer hospital stay, and they could be used for early identification of patients in need of perioperative supportive strategies.^22^

Patient-controlled propofol sedation lowers the doses required for performing endoscopic procedures, compared with sedation given by anesthetic personnel.^6^ PCS reduces the number of adverse events (oxygen desaturation or obstructed airways) and the need for interventions from personnel to act on adverse events.^6^^,^^23^ Therefore, the use of PSC is established as routine at our institution. Patients in need of advanced endoscopic procedures are often elderly and with comorbidities (in the current study, mean age was 69 years, and 75% were American Society of Anesthesiologists physical classification II or III). Although there is no criterion standard for anesthesia strategies, sedation for ERCP (without tracheal intubation) has been evaluated as equally safe as general anesthesia.^24^

Two of 5 patients displayed preoperative cognitive impairment, which is comparable to patients undergoing bronchoscopy, gastroscopy, or colonoscopy.^25^ Although cognitive impairment has previously been shown to increase the risk for poorer postoperative outcomes and adverse events,^26^ its effect on recovery is unknown. It is conceivable that patients with impaired cognition are less able to communicate their needs postprocedurally, resulting in unmet needs and negative effects on the QoR-15 domains of pain, physical comfort, emotional state, and physical independence. However, we were unable to show any such relationship with QoR-15 at any of the postprocedural sampling points.

The current study has a number of limitations. Our primary aim was to obtain indicative data on postprocedural recovery in patients undergoing advanced GIE procedures. Further studies are required to confirm the validity of these results.

Although the QoR-15 is a validated questionnaire, it may be affected by preprocedural impaired cognitive function, and many patients (both with and without impaired cognitive function preoperatively) required face-to-face or over-the-phone guidance.

The use of CIB as an indicator of preoperative cognitive impairment may be considered to be a simple screening test only and does not test neurocognitive function per se. The test is easy to use, measures both executive function and memory, and was earlier evaluated in a digital form in an endoscopy context.^25^ We can only speculate on the reasons behind the lack of a demonstrated relationship between impaired cognition and postprocedural recovery. Although 41% of patients had detectable cognitive impairment, it may not have been severe enough to limit postprocedural recovery. An alternative explanation is that many patients may have had adequate support after discharge so that they were able to facilitate good postprocedural care. Finally, the lack of a relationship may simply have been due to the limited sample size. We had no comparative data to allow sample size calculation for the secondary aim, and thus these findings may be considered as exploratory.

Nevertheless, the current study is the first, to our knowledge, to describe QoR after advanced GIE procedures and the first to study the relationship between preprocedural impaired cognitive ability and recovery. Further studies are required to confirm these findings and investigate the independent effect of impaired preoperative cognitive ability on short-term and longer-term recoveries.

In conclusion, postprocedural QoR improved significantly for a majority of the patients undergoing advanced GIE procedures with PCS. However, lack of recovery was observed in one-third of patients at postprocedural days 1 and 5. Preprocedural impaired cognitive function assessed by using the CIB test was detected in a large part of the patient group but was not an independent predictor for lack of recovery. This study highlights the need for improved strategies to increase QoR after advanced GIE procedures and that care providers should be aware of preoperative cognitive impairment and the possible effect on postoperative trajectories.

## Disclosure

All authors disclosed no financial relationships. This work was financially supported by grants from Region Östergötland County Council, Sweden, and Centre for Clinical Research Sörmland, Uppsala University, SE-Sweden.

## Figures and Tables

**Supplementary Table 1:** Strengthening the Reporting of Observational Studies in Epidemiology Statement checklist




**Supplementary Table 2:** Swedish version of the QoR-15.

**Supplementary Table 3:** Comparison of demographic and clinical data between ERCP and EUS procedures

Study group parameter	ERCP group (n = 46)	EUS group (n = 47)	*P* value∗
Age, y	69 (55-75)	69 (59-75)	.06
Female	46%	55%	.35
ASA PS class I/II/III	10%/48%/415%	16%/49%/35%	.05
Malignancy	20%	78%	<.01
Emergency procedure	61%	9%	<.01
Length of surgery, min	62 (35-91)	25 (20-34)	<.01
QoR-15 preprocedure score	112 (97-130)	124 (110-139)	.05
QoR-15 postprocedure score day 1	122 (108-135)	135 (123-143)	.02
QoR-15 postprocede score day 5	127 (119-137)	133 (126-146)	.13
QoR-15 negative change day 1	–13 (–30 to –4) (n = 17)	–7 (–13 to –2) (n = 12)	.23
QoR-15 negative change day 5	–7 (–35 to –3) (n = 10)	–8 (–21 to –3) (n = 11)	.97
PSC propofol, mg†	337 ± 179	211 ± 104	<.01
PSC alfentanil, mg‡	1.94 ± .86	1.44 ± .58	.02

Values are median (interquartile range) or mean ± SD unless otherwise indicated.

*n*, Number of participants; *ASA-PS*, American Society of Anesthesiologists Physical Status Classification; *QoR-15*, 15-item quality of recovery tool; *PCS*, patient-controlled sedation; *SD*, standard deviation.

∗ *P* = Mann-Whitney *U* test.

† PCS medication, the whole study group: propofol, mean ± SD 266 ± 155 mg.

‡ PCS medication, the whole study group: alfentanil, mean ± SD 1.62 ± .74 mg.
